# Impact of Chemotherapy for HIV-1 Related Lymphoma on Residual Viremia and Cellular HIV-1 DNA in Patients on Suppressive Antiretroviral Therapy

**DOI:** 10.1371/journal.pone.0092118

**Published:** 2014-03-17

**Authors:** Anthony R. Cillo, Supriya Krishnan, Deborah K. McMahon, Ronald T. Mitsuyasu, Michael F. Para, John W. Mellors

**Affiliations:** 1 Department of Medicine, University of Pittsburgh, Pittsburgh, Pennsylvania, United States of America; 2 Department of Biostatistics, Harvard School of Public Health, Boston, Massachusetts, United States of America; 3 Department of Medicine, University of California Los Angeles, Los Angeles, California, United States of America; 4 Department of Internal Medicine, Ohio State University, Columbus, Ohio, United States of America; University of Washington, United States of America

## Abstract

The first cure of HIV-1 infection was achieved through complex, multimodal therapy including myeloablative chemotherapy, total body irradiation, anti-thymocyte globulin, and allogeneic stem cell transplantation with a *CCR5* delta32 homozygous donor. The contributions of each component of this therapy to HIV-1 eradication are unclear. To assess the impact of cytotoxic chemotherapy alone on HIV-1 persistence, we longitudinally evaluated low-level plasma viremia and HIV-1 DNA in PBMC from patients in the ACTG A5001/ALLRT cohort on suppressive antiretroviral therapy (ART) who underwent chemotherapy for HIV-1 related lymphoma without interrupting ART. Plasma HIV-1 RNA, total HIV-1 DNA and 2-LTR circles (2-LTRs) in PBMC were measured using sensitive qPCR assays. In the 9 patients who received moderately intensive chemotherapy for HIV-1 related lymphoma with uninterrupted ART, low-level plasma HIV-1 RNA did not change significantly with chemotherapy: median HIV-1 RNA was 1 copy/mL (interquartile range: 1.0 to 20) pre-chemotherapy versus 4 copies/mL (interquartile range: 1.0 to 7.0) post-chemotherapy. HIV-1 DNA levels also did not change significantly, with median pre-chemotherapy HIV-1 DNA of 355 copies/10^6^ CD4+ cells versus 228 copies/10^6^ CD4+ cells post-chemotherapy. 2-LTRs were detectable in 2 of 9 patients pre-chemotherapy and in 3 of 9 patients post-chemotherapy. In summary, moderately intensive chemotherapy for HIV-1 related lymphoma in the context of continuous ART did not have a prolonged impact on HIV-1 persistence.

**Clinical Trials Registration Unique Identifier:**

NCT00001137

## Introduction

Effective antiretroviral therapy (ART) reduces plasma HIV-1 RNA to levels that are undetectable by FDA-approved assays, but low-level viremia and HIV-1 DNA in PBMC persist [1], [2]. The persistence of replication-competent HIV-1 in long-lived memory CD4+ T cells despite prolonged ART administration is a major obstacle to curing HIV-1 infection [3]–[5]. Nevertheless, in one HIV-infected individual, allogeneic hematopoietic stem cell transplantation (ASCT) with a *CCR5* delta32/delta32 donor resulted in the first definitive cure of HIV-1 infection [6]. This cure has generated enthusiasm for further investigation of potentially curative interventions for HIV-1, including allogeneic stem cell transplantation [7], [8] and autologous transplantation with genetically modified CD4+ T cells [9] or stem cells [10], [11]. Along these lines, the National Heart, Lung and Blood Institute (NHLBI) recently identified the possible role of hematopoietic stem cells in curative approaches for HIV-1 infection as an essential question that needs to be addressed [12].

Although there is considerable interest in stem cell-mediated interventions to achieve a cure of HIV-1, the question remains as to which components of the Berlin patient's cancer therapy were necessary to achieve a cure. Components of ASCT that may have contributed to the eradication of HIV-1 reservoirs include chemotherapy, total body irradiation, immunosuppressive drugs, allogeneic transplantation with *CCR5* delta32/delta32 donor cells and graft versus host disease. We have shown previously that myeloablative chemotherapy followed by autologous hematopoietic stem cell transplantation is not sufficient to eliminate low-level HIV-1 RNA in plasma or HIV-1 DNA in PBMC in patients on ART with <50 cps/mL of HIV-1 RNA in plasma, but changes from before myeloablative therapy to after autologous transplant were not compared in this prior study [13], thus the impact chemotherapy alone is undefined. Intensive chemotherapy is known to cause significant depletion of circulating CD4+ T cells [14]–[16], which could reduce levels of plasma viremia or HIV-1 DNA in PBMC in the context of uninterrupted ART by killing HIV-infected cells. To investigate this possibility, we measured HIV-1 levels in plasma and PBMC samples, before and after chemotherapy, in 9 patients who underwent moderately intensive chemotherapy for HIV-1 related lymphoma and who remained on continuous ART throughout the sampling period.

## Methods

NWCS 334 was a retrospective study of HIV-1-infected patients in the ACTG A5001: AIDS Clinical Trials Group Longitudinal Linked Randomized Trials (ALLRT) cohort who received moderately intensive chemotherapy for the treatment of HIV-1 related lymphoma, who continued on ART pre- and post-chemotherapy, and who maintained suppressed plasma HIV-1 RNA<50 copies/mL (Roche Amplicor HIV Monitor assay versions 1.0/1.5; Branchburg, NJ). The ALLRT parent study is registered at ClinicalTrials.gov under the unique identifier NCT00001137, and the rationale, design, and baseline characteristics of the overall cohort have been previously described [17]. The University of Pittsburgh Institutional Review Board approved the parent study (ALLRT), which allowed participants to contribute samples for future use in ACTG-approved research. Patients gave written informed consent for the information to be obtained from their clinic records as part of the ALLRT study, and this information was kept confidential at each ALLRT site. Patient information was anonymized and de-identified prior to analysis.

Stored plasma samples were evaluated for HIV-1 RNA using two-step real-time quantitative PCR assays with two different primer/probe sets targeting HIV-1 *gag* or *integrase* sequences using previously described assay conditions with single-copy sensitivity (limit of detection<1 copy/mL of plasma) [13], [18]. Levels of total HIV-1 DNA (limit of quantification = 5 copies/sample) and 2-long terminal repeat circles (2-LTRs; limit of quantification = 7.5 copies/sample) in PBMC were assayed as described previously [13] and were run in parallel with positive and negative controls from the Virology Quality Assurance Laboratory (Rush University). HIV-1 DNA quantitative PCR (qPCR) data were normalized per 10^6^ CD4+ T cells using qPCR for the *CCR5* gene [19] and the percent CD4+ T cells. The percent CD4+ T cells was available from the A5001/ALLRT database (N = 8) or was determined by standard flow cytometry in the Pitt Virology Support Laboratory (N = 1).

Statistical analysis using McNemar's Test was applied to determine if the proportion of patients with plasma viremia at undetectable levels pre-chemotherapy was significantly different from the proportion of patients with undetectable levels post-chemotherapy. A non-parametric sign test was used to determine if there was a significant difference between CD4+ and CD8+ cell counts pre- and post-chemotherapy, and between HIV-1 DNA copies per 10^6^ CD4+ T cells pre- and post-chemotherapy.

## Results

A total of 40 patients in the A5001/ALLRT cohort were diagnosed with HIV-1 related lymphoma, 18 had plasma HIV-1 RNA<50 cps/mL pre- and post-chemotherapy with uninterrupted ART, and 10 of these 18 had plasma and PBMC samples available pre- and post-chemotherapy for further analysis. To confirm the efficiency of qPCR amplification for HIV-1 RNA, plasma samples from prior to the initiation of ART in these 10 patients were tested and results were compared to the FDA-approved Roche Amplicor assay. HIV-1 RNA in pre-ART samples from 9 of 10 subjects amplified efficiently by qPCR, and longitudinal samples from these 9 patients were studied. The relevant clinic characteristics of the study patients are shown in Table 1. All patients were males diagnosed with HIV-1 related Hodgkin's (HL; N = 3) or Non-Hodgkin's lymphoma (NHL; N = 6).

**Table 1:** Characteristics of the 9 patients studied from the ALLRT cohort.

Patient ID Number	Sex	Race/Ethnicity	Age at Lymphoma Diagnosis	Lymphoma Diagnosis	Chemotherapy Administered	CD4+ T cell Counts (cells/mm^3^)		Time from Pre- to Post-Chemotherapy Sampling (Days)
						Pre-Chemo	Post-Chemo	
1	Male	White	69	NHL	R-CHOP	296	380	823
2	Male	Black	69	HL	ABVD	205	122	225
3	Male	White	45	NHL	CHOP	450	453	237
4	Male	White	46	NHL	R-CHOP	397	315	685
5	Male	Hispanic	61	HL	ABVD	130	162	181
6	Male	Black	36	NHL	ABVD	848	513	784
7	Male	White	54	NHL	CHOP	238	199	90
8	Male	Hispanic	35	HL	ABVD	524	662	580
9	Male	Hispanic	48	NHL	CHOP	232	251	285

NHL: Non-Hodgkin's Lymphoma; HL: Hodgkin's Lymphoma; R-CHOP: rituximab, cyclophosphamide, doxorubicin, vincristine, prednisone; ABVD: doxorubicin, bleomycin, vinblastine, dacarbazine; CHOP: cyclophosphamide, doxorubicin, vincristine, prednisone.

All 9 patients received moderately intensive chemotherapy for lymphoma consisting of doxorubicin, bleomycin, vinblastine and dacarbazine (ABVD) in 4 patients, cyclophosphamide, doxorubicin, vincristine, prednisone (CHOP) in 3 patients, and CHOP with rituximab in 2 patients (Table 1). The number of days between pre- and post-chemotherapy sampling varied, with a median of 285 days between samples (interquartile range: 225 to 685 days). Median CD4+ count pre-chemotherapy was 296 cells/μL (median %CD4 = 20.0) and was 315 cells/μL (median %CD4 = 20.0) post-chemotherapy; median CD8+ count pre-chemotherapy was 803 cells/μL (median %CD8 = 50.0%) and 693 cells/μL (median %CD8 = 49.0%) post-chemotherapy. Differences in the CD4+ and CD8+ cell counts between pre- and post-chemotherapy time points were not statistically significant.

**Figure 1:**
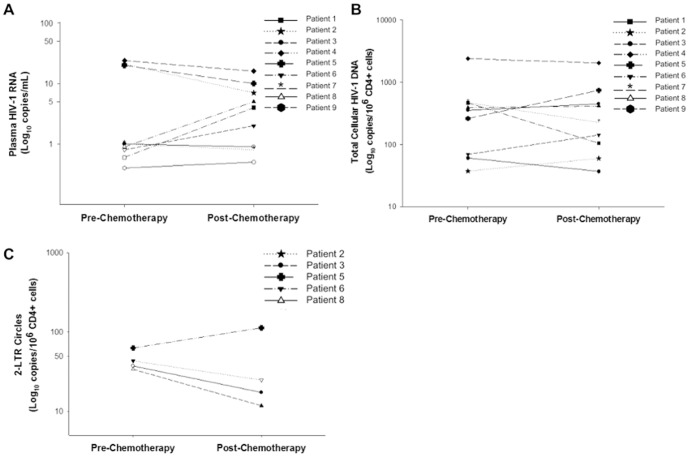
Quantification of HIV-1 from plasma and PBMC pre- and post-chemotherapy. **A**) Plasma HIV-1 RNA decreased for 5 patients, increased for 3 patients, and was below the limit of detection at both time points for 1 patient. Median viral load pre-chemotherapy was 1 copy/mL versus 4 copies/mL post-chemotherapy. Open symbols indicate undetectable samples. **B**) Total HIV-1 DNA levels per 10^6^ CD4+ cells decreased in 4 patients and increased in 5 patients. Median HIV-1 DNA levels per 10^6^ CD4+ cells decreased from 355 to 228 copies per 10^6^ CD4+ cells from pre- to post-chemotherapy. **C**) 2-LTRs were detectable pre-chemotherapy in 2 of 9 patients versus detectable in 3 of 9 post-chemotherapy. Patients with undetectable 2-LTRs at both time points are not shown. Open symbols indicate undetectable samples.

All subjects received ART throughout their chemotherapy and post-chemotherapy follow-up. Low-level plasma HIV-1 RNA (Figure 1A), as determined by qPCR with single-copy sensitivity [13], did not show a consistent pattern of change from pre- to post-chemotherapy time points. Plasma viremia decreased in 5 patients (median decrease = 8 copies/mL), increased in 3 (median increase = 3 copies/mL), and remained below the LOD in one patient (Figure 1A). The median HIV-1 plasma RNA pre-chemotherapy was 1 copy/mL, and the median post-chemotherapy was 4 copies/mL. A total of 4 patients had undetectable plasma HIV-1 RNA pre-chemotherapy and 3 had undetectable plasma HIV-1 RNA post-chemotherapy. There was no significant difference in the proportion of patients with undetectable plasma HIV-1 RNA before versus after chemotherapy (p = 0.6, McNemar's test).

Levels of total HIV-1 DNA and 2-LTRs in PBMC (Figures 1B and 1C) were evaluated for changes between pre- and post-chemotherapy time points. Total HIV-1 DNA was detectable in all 9 patients pre- and post-chemotherapy and showed no consistent pattern of change. The median total HIV-1 DNA level pre-chemotherapy was 355 (interquartile range: 70.0 to 469) copies/10^6^ CD4+ T cells versus 228 (interquartile range: 106 to 452) copies/10^6^ CD4+ cells post-chemotherapy (p = 1.0, sign test). Total HIV-1 DNA levels per 10^6^ CD4+ cells decreased in 4 patients and increased in 5 patients between pre- and post-chemotherapy time points. 2-LTRs were detectable in only 4 of 9 subjects at either time point, with 2-LTRs increasing in 1 patient post-chemotherapy, becoming detectable (from below the limit of quantification) in 2 subjects, and becoming undetectable in 1 subject.

## Discussion

In this small initial study (N = 9), we found no significant differences between pre- and post-chemotherapy levels of HIV-1 RNA in plasma and HIV-1 DNA in PBMC from patients receiving chemotherapy for HIV-1 related lymphoma. The absence of a durable effect on plasma viremia following chemotherapy suggests that there was not a reduction in the number of infected cells that can produce virus. Although much of the HIV-1 DNA that persists despite ART has deletions or is hypermutated [20]–[22], the lack of a reduction in HIV-1 DNA levels is also consistent with chemotherapy not causing a sustained reduction in infected cell number.

One reason why chemotherapy would not impact HIV-1 persistence is that some subpopulations of CD4+ T cells may be resistant to chemotherapeutic agents. In this regard, Turtle et al. have described a population of CD8+ T cells in peripheral blood that survive intensive chemotherapy [23], and Casorati et al. have described a population of bone marrow resident CD4+ T cells that survive conditioning chemotherapy for autologous transplantation [24]. Importantly, the latent reservoir of HIV-1 resides within the resting memory CD4+ T cell population [3], and while chemotherapy significantly depletes CD4+ T cells in the periphery [8], [24], resting memory CD4+ T cells may be more resistant to cytotoxic chemotherapy because of their quiescent state [15]. In addition, CD4+ T cells that survive cytoreductive chemotherapy and harbor HIV-1 DNA are likely to proliferate in response to chemotherapy-induced lymphopenia through IL-7 mediated homoestatic proliferation [25], [26]. Hence, although chemotherapy may kill some HIV-infected cells, those that survive could repopulate HIV-1 reservoirs through cell proliferation in response to lymphopenia.

Limitations of our study are the small sample size and long interval (median 285 days) between pre- and post-chemotherapy samples. As a consequence, transient reductions in low-level viremia and HIV-infected CD4+ T cells from chemotherapy could have been missed, as could have restoration of viremia and HIV-infected cells through proliferation of surviving CD4+ T cells. Nevertheless, the findings from this current study are similar to those found in a cross-sectional study of 10 patients on suppressive ART, where HIV-1 RNA in plasma and HIV-1 DNA in PBMC remained detectable following myeloablative chemotherapy and autologous stem cell transplantation [13]. The current study adds to this prior post-transplant cross-sectional study by comparing pre- and post-chemotherapy levels of HIV-1 persistence.

The failure of either moderately intensive or myeloablative chemotherapy to have a sustained effect on HIV-1 persistence points to the importance of allogeneic transplantation with a *CCR5* delta32 homozygous donor in achieving the first definitive cure of HIV-1 infection [6]. For HIV-1 to infect target cells, CD4 and one of two major coreceptors, either CCR5 or CXCR4, must be expressed on the cell surface [27], [28]. A 32 base pair deletion (*CCR5* delta32) provides resistance to CCR5 tropic HIV-1 [29], [30], and is present in 2–5% of persons from Europe, the Middle East and the Indian subcontinent [31]. Complete replacement of the Berlin patient's immune system by allogeneic cells with the *CCR5* delta32 mutation was likely critical in eradicating HIV-1 reservoirs. Recently, the elimination of HIV-1 DNA from PBMC in 2 patients who received ART throughout reduced-intensity allogeneic transplantation with CCR5 wild-type donors has been reported [7], confirming the importance of allogeneic transplantation in eliminating evidence of HIV-1 persistence in blood. The recent report of viral rebound in both of these patients 3–8 months after cessation of ART indicates that HIV-1 reservoirs were not eliminated by allogeneic transplantation and that the CCR5 wild-type donor cells supported HIV-1 replication in the absence of ART [32].

In summary, this study provides evidence that chemotherapy alone does not have a sustained impact on HIV-1 persistence in patients on ART and that future therapeutic interventions to reduce or eliminate HIV-1 reservoirs will need to have greater specificity for HIV-infected cells.
